# Intermittent Fasting Reshapes the Gut Microbiota and Metabolome and Reduces Weight Gain More Effectively Than Melatonin in Mice

**DOI:** 10.3389/fnut.2021.784681

**Published:** 2021-11-24

**Authors:** Jingliang Liu, Yifan Zhong, Xin M. Luo, Yanfei Ma, Jianxin Liu, Haifeng Wang

**Affiliations:** ^1^College of Animal Science, Zhejiang University, The Key Laboratory of Molecular Animal Nutrition, Ministry of Education, Hangzhou, China; ^2^Department of Biomedical Sciences and Pathobiology, Virginia Tech, Blacksburg, VA, United States

**Keywords:** intermittent fasting, melatonin, liver, intestinal morphology, gut microbiota, metabolites

## Abstract

**Background:** Intermittent fasting (IF) can reduce energy intake and body weight (BW). Melatonin has many known functions, which include reducing appetite and preventing excessive weight gain.

**Objective:** This study aimed to investigate the effects of IF on body fat and the gut microbiota and metabolome as well as a potential interaction with melatonin.

**Methods:** Male C57BL/6J mice (23.0 ± 0.9 g, 6 wk old) were randomly assigned into four groups (12 mice/group): control (C), intermittent fasting (F), melatonin (M), and intermittent fasting plus melatonin (MF). The C and M groups mice were provided with *ad libitum* access to food and water, while the F and MF groups underwent alternative-day feed deprivation (15 cycles total). Melatonin was administered in the drinking water of the M and MF groups. Blood, epididymal fat, liver tissue, and intestinal tissue and contents were collected for lab measurements, histology, and microbiota and metabolome analysis. Main effects and interactions were tested by 2-factor ANOVA.

**Results:** IF significantly reduced BW gain and serum glucose, total cholesterol (TC) and triglyceride (TG) levels. Adipocyte size significantly decreased with IF, then the number of adipocytes per square millimeter significantly increased (*P* < 0.05). Compared to the C group, the M and MF groups had significantly higher serum melatonin levels (17 and 21%, respectively), although melatonin monotherapy had no effect on serum parameters and adipocytes. There was no interaction between IF and melatonin on BW gain and serum parameters except for on adipocyte area and number per square millimeter, *Bacteroidetes* and *Akkermansia* bacterial abundance, and the levels of the intestinal metabolites alanine, valine and isoleucine. IF changed the intestinal microbiota structure, with the F and MF groups clearly separating from the C and M groups. Metabolomic analysis showed that there was obvious separation between all four groups.

**Conclusions:** IF, but neither melatonin nor the interaction between IF and melatonin, could alter intestinal microbiota and metabolism and prevent obesity by reducing BW gain, serum glucose, TC, and TG, and adipocyte size in mice.

## Introduction

Agricultural developments and improve living standards have contributed to a rise in obesity. Obesity has a physiologic influence on organs such as the liver, visceral fat, and the circulatory system (1). It increases the risk of diseases such as diabetes mellitus (2), cardiovascular disease (2), and several types of cancers (3). Imbalance between energy intake and expenditure is the main cause of obesity (4). Intermittent fasting (IF) is a dietary energy restriction method that reduces energy intake and thereby reduces obesity. From an evolutionary point of view, fasting is a natural phenomenon that humans and lower organisms were regularly exposed to. Humans, especially in some religious groups (5), have practiced IF for centuries, and IF has been shown to improve body composition and health (6, 7). IF reduces body weight, fat mass and caloric intake comparable to a low-fat diet, and glucose and insulin tolerance can be altered by IF (8). Moreover, IF extends overall lifespan and reduces the development of aging-related diseases, including diabetes, cardiovascular disease and neurodegenerative diseases (9). Previous studies have shown that altering caloric intake or meal timing can delay the occurrence and development of disease and improves the health and lifespan of most organisms (10, 11). The potential physiologic processes involved in IF include periodic changes in the source of metabolic fuel, the promotion of repair mechanisms, and the optimization of healthy energy utilization by cells and body (12).

Melatonin is a biochemical hormone secreted by the pineal gland that inhibits melanin formation (13). Melatonin-related enzymes and receptors are found in almost every tissue and cell (14), suggesting that melatonin has a wide range of functions. As a powerful antioxidant, melatonin can control the survival and differentiation of immune cells, scavenge free radicals, and increase the activity of antioxidant enzymes, thereby reducing inflammation and resisting oxidative stress (15). Melatonin can also protect the liver from oxidative stress-induced damage (16).

Previous studies have shown that melatonin drives the body's energy balance toward reducing food intake, increases energy consumption by burning brown adipose tissue, and prevents excessive weight gain (17). Melatonin reduced body weight, liver steatosis and low-grade inflammation while improving insulin resistance in high fat diet (HFD)-fed mice (18). Melatonin levels in the gut are at least 400 times higher than in the pineal gland, and 10–100 times higher than in the blood (19), suggesting that melatonin may play an important role in normal gut function. The role of melatonin in the prevention and treatment of intestinal diseases has been borne out by prior literatures (15, 20). Melatonin can also serve as a mediator of microbial metabolism, circadian rhythms, and intestinal mucosal immune cells (20). Melatonin treatment significantly changed the composition of the gut microbiota in mice fed a HFD (18). Gut microbiota, directly affects the gastrointestinal (GI) tract, liver, skin, and central nervous system, and participates in the digestion and absorption of nutrients (21, 22). Due to the interrelationship between melatonin and the gut microbiota, melatonin has been hypothesized to be involved in communication between the gut tissue and the intestinal microbiota (15). Gut bacteria has been found to recognize and respond to melatonin signals in the intestine *via* melatonin binding sites (23), supporting this relationship.

Lots of studies on obese people showed IF benefits for preventing metabolic disorder, however, it is deserved to find what effects IF may exert on metabolism of people on a popular normal diet. Melatonin, as a hormone regulating circadian rhythm, can also affect body weight gain and intestinal microbiota. It is interesting to explore if the interaction exits between the IF and melatonin. This study aimed to investigate the potential interaction of IF and oral melatonin on body weight, blood indices, intestinal and liver morphology, and intestinal microbiota and metabolites in an experimental mouse model.

## Materials and Methods

### Animals and Experimental Design

Male C57BL/6J mice (23.0 ± 0.9 g, 6 weeks of age) were obtained from Shanghai SLAC Laboratory Animal Co., Ltd (Shanghai, China). All animals were maintained under standardized conditions (23 ± 1°C; 12-h light-dark cycle). All animal procedures were performed in full accordance with the “Regulation for the Use of Experimental Animals” of Zhejiang Province, China. This study was specifically approved by the Animal Care and Use Committee of Zhejiang University (ETHICS CODE Permit no. ZJU20170529). Animals were fed commercial feed (#P1101F, Slacom, Shanghai, China) composed of fish meal, wheat, corn, soybean meal, wheat bran, vitamin, mineral and amino acids that contained at least 20.5% crude protein, 4% crude fat, 1.32% lysine, and 0.78% methionine + cystine, and ≤ 5% crude fiber, and ≤ 8% crude ash. After acclimatization for 10 days, mice were randomly assigned into four groups (12 mice/group): control (C), intermittent fasting (F), melatonin (M), and intermittent fasting plus melatonin (MF). The C and M groups mice were provided with *ad libitum* access to food and water, while the F and MF groups underwent alternative-day feed deprivation (15 cycles total). Melatonin was administered in the drinking water of the M and MF groups. Due to the experimental design, we are not able to determine the melatonin intake for each mouse; instead, melatonin was titrated to an averaged dose of 10 mg/kg body weight and provided in the drinking water. The melatonin water was prepared daily and kept in a normal bottle with an aluminum foil cover to prevent light-induced melatonin degradation.

Body weight and food/water intake were measured daily. The mice were euthanized at the end of the trial with an intraperitoneal injection of pentobarbital sodium (50 mg/kg body weight), and blood and tissue samples were collected.

### Serum Biochemical Analysis

Blood samples were centrifuged at 3,000 × g for 10 min at 4°C to produce serum samples. The levels of serum glucose, triglyceride (TG), total cholesterol (TC), low density lipoproteins cholesterol (LDL-C), and high density lipoproteins cholesterol (HDL-C) were quantified using corresponding ELISA kits (no. F006, no. A110-1, no. A111-1, no. A113-1, and no. A112-1, respectively; Nanjing Jiancheng Bioengineering Institution, Nanjing, China). Serum levels of alanine aminotransferase (ALT) (no. C009) and aspartate aminotransferase (AST) (no. C010) were measured using kinetics-based assays with commercially available kits (Nanjing Jiancheng Bioengineering Institution, Nanjing, China) and an automatic biochemistry analyzer (SELECTA XL; Vital Scientific, Newton, MA, USA) according to protocols provided by the manufacturers. Serum melatonin and insulin levels were measured using ELISA kits (no. H256-1-2 and no. H203-1-2, respectively; Nanjing Jiancheng Bioengineering Institution, Nanjing, China).

### Hematoxylin-Eosin (H&E) Staining

H&E staining was performed as previously described (24). Epididymal adipose, liver, ileal, and colonic samples were fixed, dehydrated, and paraffin embedded. The sections were prepared and subsequently stained with H&E. Photomicrographs were obtained using an optical microscopy system (Olympus Corporation, Tokyo, Japan). Quantitative measurement of adipocyte count, ileal villi height, ileal crypt depth, and colonic fold height were conducted with ImageJ (National Institutes of Health, Bethesda, MD, USA). Adipocytes area was calculated using at least three histological sections and a total of 300 adipocytes per mouse. A pathologist evaluated the liver slides.

### Transmission Electron Microscopy (TEM)

TEM of liver tissues was performed as previously described (25, 26). The specimen was sectioned using a LEICA EM UC7 ultratome, and sections were stained with uranyl acetate and alkaline lead citrate. Section were visualized with a Hitachi H-7650 TEM.

### 16S rDNA Gene Analysis

The entire intestinal contents were collected and used for bacterial 16S rDNA sequencing. High-resolution 16S rDNA genes of the intestinal bacterial flora were analyzed using a previously described method with modifications (27). DNA was extracted from the intestinal contents using the Qiagen DNA Kit (51640, Germany) according to the manufacturer's instructions. The selected region of 16S rDNA amplification was the V3–V4 region PCR products were quantified using Qubit (Invitrogen, USA). An Illumina NovaSeq PE250 (Illumina, San Diego, USA) was used for on-board sequencing, followed by bioinformatics analysis. Chimeric sequence detection and *de novo* operational taxonomic units (OTU) picked up with 0.97 identities were performed using Usearch (version 7.0) and UPARSE (http://drive5.com/uparse/), respectively (28). Alpha/beta diversity and the relative abundance of bacteria at the phylum and genus level were analyzed with QIIME1 (V1.9.1). Principal coordinates analysis (PCoA) of Weighted-Unifrac distance was used to visualize the bacteria communities of each of the four groups. PICRUSt was used to predict the functional composition of the metagenome, and the Kyoto Encyclopedia of Genes and Genomes (KEGG) Orthology functional database was used as reference for this prediction (29). Linear discriminant analysis (LDA) effect size (LEfSe) was used to estimate the effect of each component metabolic pathway on the difference, and to find out the metabolic pathway that had a significant difference on the sample division (The default screening condition is LDA >2).

### Metabolomic Analysis

About 50 mg sample with 500 μL pre-cold extraction mixture [methanol/chloroform (v:v) = 3:1] and 10 μL internal standard (L-2-Chlorophenylalanine, 1 mg/mL stock) were added into a 2 mL tube. Then the sample was vortexed for 30 s and homogenized with ball mill for 4 min at 35 Hz, followed by ultrasonication for 5 min on ice water. The mixture was extracted and centrifuged at 13,300 × g for 15 min at 4°C. Supernatant was collected and analyzed using gas chromatography coupled with a time-of-flight mass spectrometer (GC-TOF-MS) according to a previous method (30), which did not distinguish between positive and negative, and the results did not distinguish between polarity and non-polarity. The LECO-Fiehn Rtx5 database was used to identify metabolites by matching the mass spectrum and the retention index (31). Peaks detected in less than half of the QC samples or with a RSD >30% in the QC samples was removed (32). Follow-up analysis of the obtained data was performed using existing methods (33). The final dataset containing the information of peak number, sample name and normalized peak area was imported into MetaboAnalyst 4.0 (https://www.metaboanalyst.ca/) for multivariate analysis. Partial least squares discrimination analysis (PLS-DA) and *t*-test were performed between the two groups, with a false discovery rate (FDR) adjusted *P* < 0.05 and variable importance in projection (VIP) > 1.5 used to identify a significant different in metabolites. Commercial databases including KEGG (http://www.genome.jp/kegg/) were used for pathway enrichment analysis.

### Statistical Analysis

Statistical analysis was performed using SAS software (SAS9.04, Cary NC, USA). All data are presented as mean ± standard error (SE). Significant differences were identified using 2-factor ANOVAs with a general linear model for this 2 x 2 factorial experiment and IF x melatonin interaction was tested, followed by Duncan's multiple range tests. A *P*-value of <0.05 was considered statistically significant.

## Results

### Growth Performance

**Figure 1:**
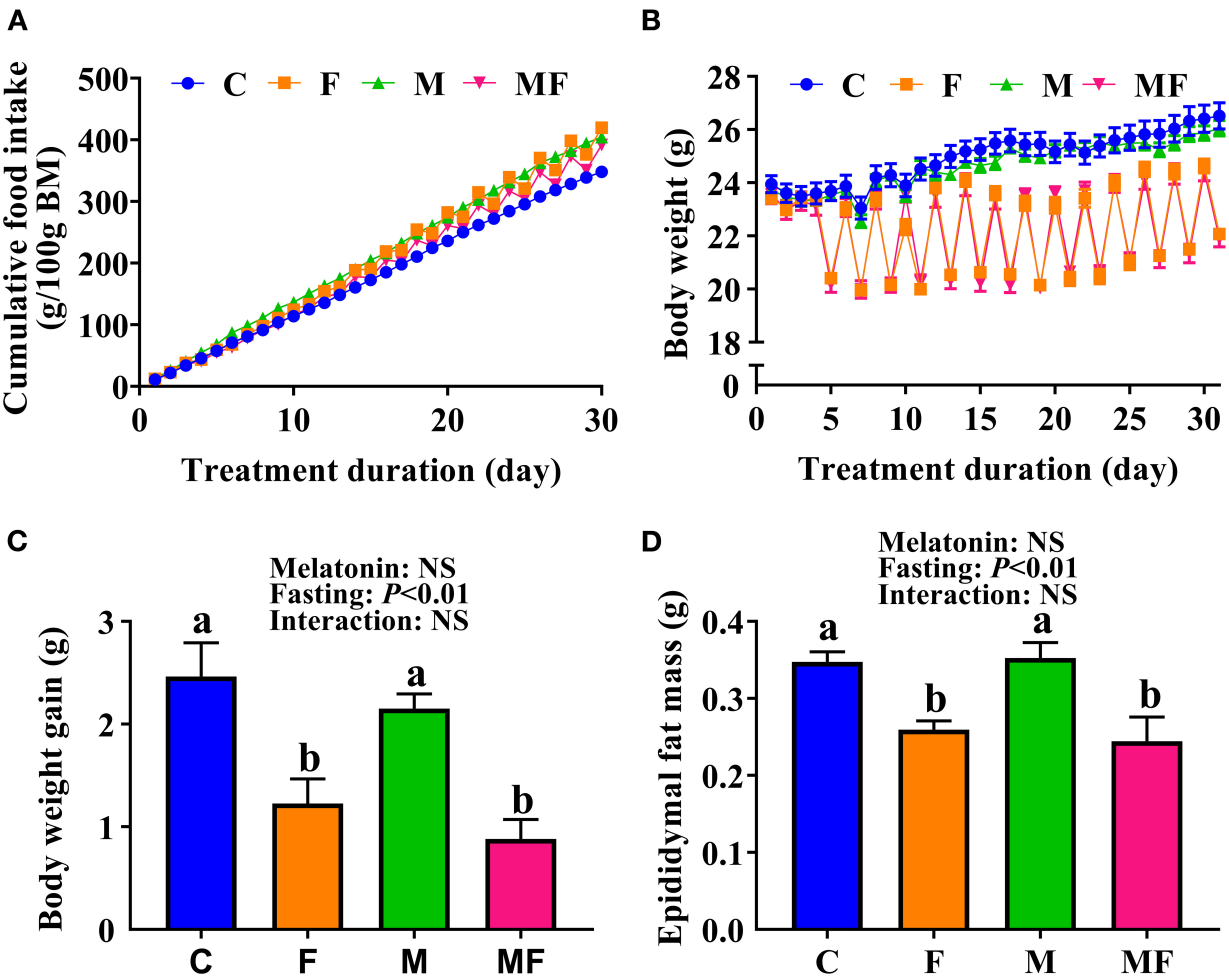
The effects of intermittent fasting and melatonin supplementation on food intake and body weight gain in mice. **(A)** Cumulative food intake. **(B)** Body weight. **(C)** Weight gain after treatment for 30 days. **(D)** Epididymal fat mass of mice at the end of the experiment. Data are presented as mean ± SEM, *n* = 12; Labeled means without a common letter differ (*P* < 0.05), NS: *P* ≥ 0.05. C, control; F, intermittent fasting; M, melatonin; MF, intermittent fasting plus melatonin. The C and M mice were fed *ad libitum*, while the F and MF groups underwent alternative-day feed deprivation. The M and MF groups mice were supplemented with melatonin at a dose of 10 mg/kg body weight by drinking.

Compared with the C group, IF and melatonin did not affect cumulative food intake (Figure 1A). However, IF (F and MF) led to significantly lower BW (Figure 1B) and significantly lower BW gain (50 and 64% lower than C, respectively, Figure 1C). Food intake after fasting for 24 h sharply increased (Supplementary Figure 1A). When the mice fasted, their water intake was also lower than normal (Supplementary Figure 1B). The average dose of melatonin over the study period was shown in Supplementary Figure 1C (one cycle means a non-fasting day and a fasting day), and confirms that the average daily melatonin intake was close to 10 mg/kg. In addition, epididymal fat mass in the F and MF groups was significantly lower than in the C group (25 and 30%, respectively; Figure 1D). Melatonin alone, and the use of melatonin with fasting did not have a significant impact on BW and epididymal fat mass (*P* > 0.05).

### Serum Indices

**Figure 2:**
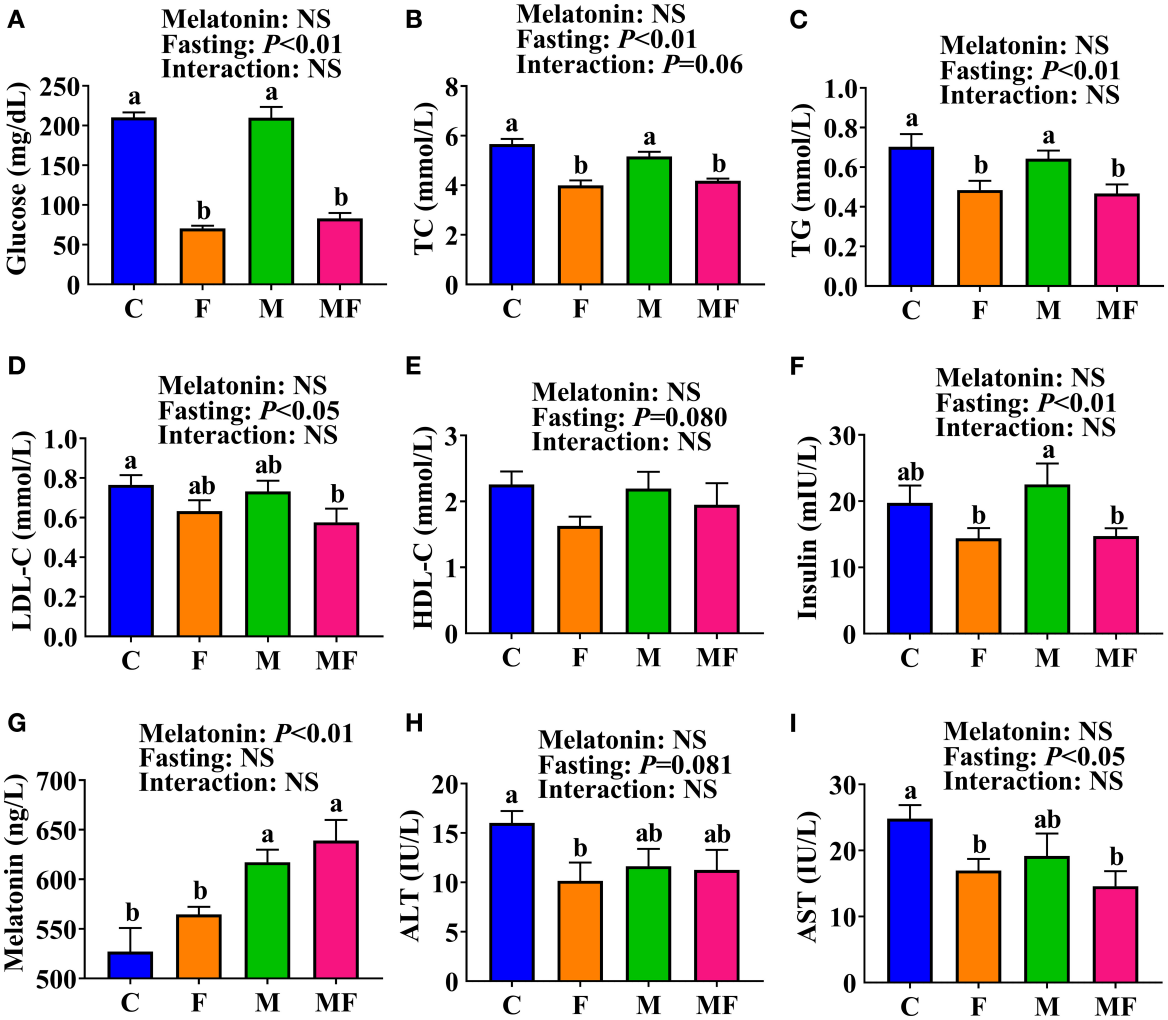
The effects of intermittent fasting and melatonin supplementation on serum indexes in mice. **(A)** Glucose; **(B)** TC; **(C)** TG; **(D)** LDL-C; **(E)** HDL-C; **(F)** Insulin; **(G)** Melatonin; **(H)** ALT; **(I)** AST. Data are presented as mean ± SEM, *n* = 8~12. Labeled means without a common letter differ (*P* < 0.05), NS: *P* ≥ 0.05. C, control; F, intermittent fasting; M, melatonin; MF, intermittent fasting plus melatonin. The C and M mice were fed *ad libitum*, while the F and MF groups underwent alternative-day feed deprivation. The M and MF groups mice were supplemented with melatonin at a dose of 10 mg/kg body weight by drinking. ALT, alanine aminotransferase; AST, aspartate aminotransferase; HDL-C, high density lipoproteins cholesterol; LDL-C, low density lipoproteins cholesterol; TC, total cholesterol; TG, triglyceride.

The F and MF groups had significantly lower serum glucose (66 and 61% lower, respectively, *P* < 0.05, Figure 2A), TC (29 and 26% lower, respectively, *P* < 0.05, Figure 2B) and TG (31 and 34% lower, respectively, *P* < 0.05, Figure 2C) levels than the C group. There was no significant difference in serum glucose, TC, or TG between the M and C groups, or between the MF and F groups. However, the MF group had significantly lower serum glucose (61% lower, *P* < 0.05, Figure 2A), TC (19% lower, *P* < 0.05, Figure 2B) and TG (27% lower, *P* < 0.05, Figure 2C) than the M group. Compared with the C group, MF group mice had significantly lower serum LDL-C (25% lower, *P* < 0.05, Figure 2D). No significant difference was observed in serum HDL-C (Figure 2E).

Serum insulin level was significantly lower in the F (36% lower) and MF (35% lower) groups than the M group (*P* < 0.05, Figure 2F). Serum melatonin level was significantly higher in the M and MF group than in the C (17 and 21% higher, respectively, *P* < 0.05) and F (9 and 13% higher, respectively, *P* < 0.05) groups (Figure 2G). Compared with the C group, the F group had significantly lower serum ALT (37% lower, *P* < 0.05, Figure 2H) and AST (32% lower, *P* < 0.05, Figure 2I), whereas the MF group had significantly lower serum AST (41% lower, *P* < 0.05) and an equivalent serum ALT. There was little interaction between IF and melatonin in serum parameters (*P* > 0.05).

### Intestinal Morphology

The morphology of ileal and colonic tissues was visualized with H&E staining and shown in Supplementary Figures 2A,B, respectively. Compared with the C group, the F group mice had significantly higher villus height (13% increase, *P* < 0.05) and villus height/crypt depth ratio (20% increase, *P* < 0.05, Supplementary Figure 2C). The villus height/crypt depth of the MF group was also significantly higher than that of the C group (15% increase, *P* < 0.05). There were no significant differences in ileal crypt depth identified. There were no significant differences in colonic fold length between groups (*P* > 0.05, Supplementary Figure 2D). No significant interaction between IF and melatonin was found on villus height, crypt depth and colonic fold length (*P* > 0.05).

### Adipocyte Morphology in Epididymal White Adipose Tissue

**Figure 3:**
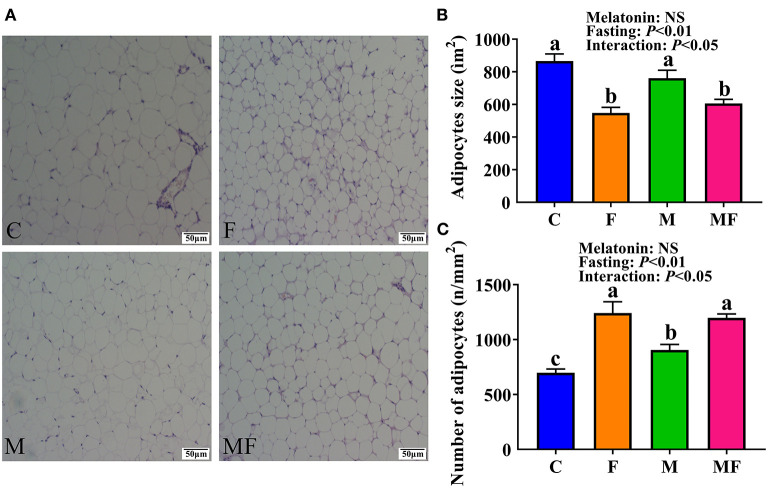
The effects of intermittent fasting and melatonin supplementation on adipocytes morphology in mice. **(A)** Representative H&E staining of epididymal WAT sections. Scale bar: 50 μm; **(B)** quantification of adipocyte size; **(C)** quantification of adipocyte number per square millimeter. Data are presented as mean ± SEM, *n* = 6. Labeled means without a common letter differ (*P* < 0.05), NS: *P* ≥ 0.05. C, control; F, intermittent fasting; M, melatonin; MF, intermittent fasting plus melatonin. The C and M mice were fed *ad libitum*, while the F and MF groups underwent alternative-day feed deprivation. The M and MF groups mice were supplemented with melatonin at a dose of 10 mg/kg body weight by drinking.

Histologic analysis of the epididymal white adipose tissue (eWAT) revealed an increase in the number of multilobular adipocytes in IF mice (Figure 3A), a typical characteristic of beige adipocytes. F and MF group mice had a significantly smaller adipocyte size (37 and 20% decrease, respectively, *P* < 0.05, Figure 3B), but a significant increase in adipocyte number per square millimeter (78 and 32% increase, respectively, *P* < 0.05, Figure 3C), compared with the corresponding non-fasting group (C and M, respectively). M group mice had a significantly higher number of adipocytes per square millimeter than C group mice (30% increase, *P* < 0.05). The interaction between fasting and melatonin had significant effects on adipocytes size and number (*P* < 0.05, Figures 3B,C).

### Morphologic Structure of Liver Tissue

**Figure 4:**
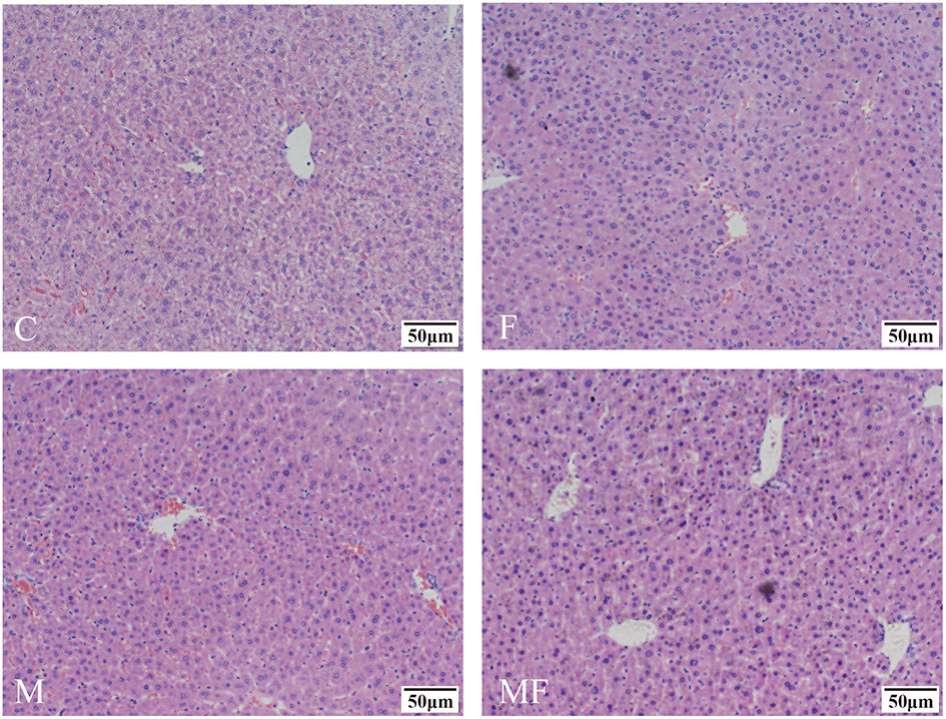
Representative H&E samples showing differences in liver morphology in control mice vs. those with intermittent fasting and/or melatonin treatment. Scale bar: 50 μm. C, control; F, intermittent fasting; M, melatonin; MF, intermittent fasting plus melatonin. The C and M mice were fed *ad libitum*, while the F and MF groups underwent alternative-day feed deprivation. The M and MF groups mice were supplemented with melatonin at a dose of 10 mg/kg body weight by drinking.

**Figure 5:**
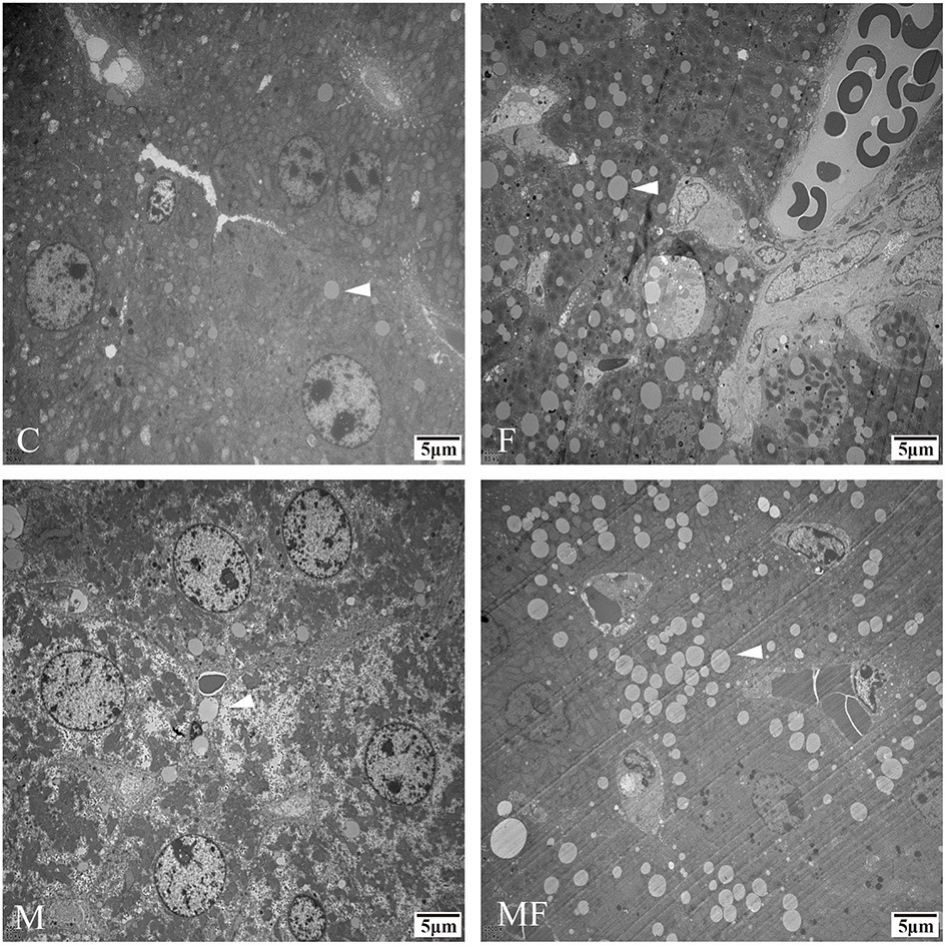
Representative TEM images depicting the number of lipid droplets in the livers of control mice vs. those with intermittent fasting and/or melatonin treatment. Scale bar: 5 μm. Images of the fasting groups (F and MF) revealed a great number of lipid droplets (white arrows). C, control; F, intermittent fasting; M, melatonin; MF, intermittent fasting plus melatonin. TEM, Transmission electron microscopy.

Liver tissue morphology was normal in all groups, without obvious signs of inflammation or lesions (Figure 4). However, sections of the liver assessed with TEM showed a greater number of lipid droplets in the fasting groups (F and MF) compared with the C and M groups (Figure 5).

### Analysis of Intestinal Microbiota

**Figure 6:**
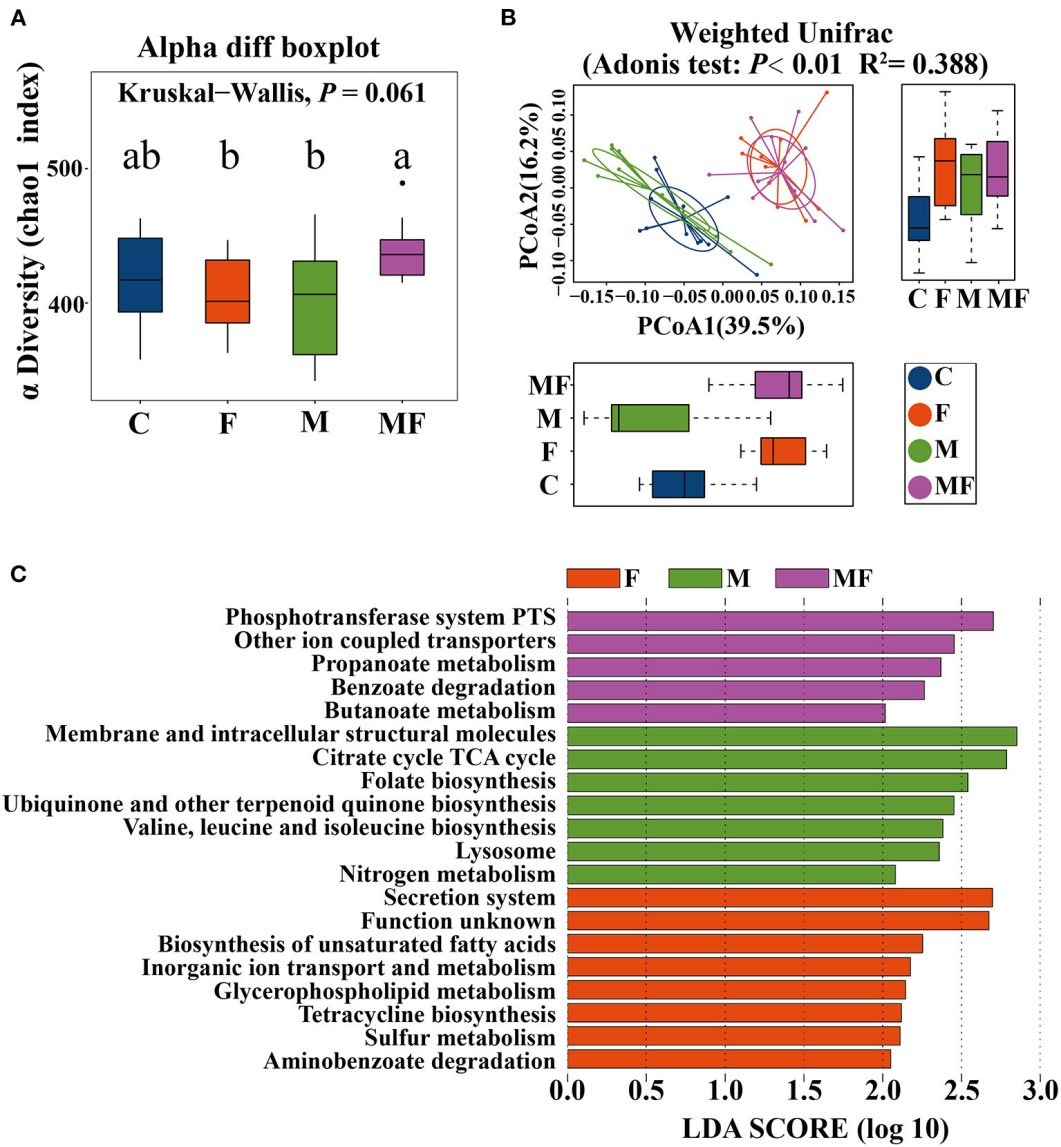
The effects of intermittent fasting and melatonin supplementation on intestinal microbiota in mice. **(A)** Differences in the α diversity index between groups (Chao 1 diversity index); **(B)** principal Coordinates Analysis (PCoA) of intestinal microbiota (β diversity); **(C)** LEfSe (LDA Effect Size) analysis of KEGG pathway enrichment in different groups, *n* = 12; Labeled means without a common letter differ (*P* < 0.05). C, control; F, intermittent fasting; M, melatonin; MF, intermittent fasting plus melatonin. The C and M mice were fed *ad libitum*, while the F and MF groups underwent alternative-day feed deprivation. The M and MF groups mice were supplemented with melatonin at a dose of 10 mg/kg body weight by drinking.

The rarefaction curve of observed species (Supplementary Figure 3A) and Chao 1 index (Supplementary Figure 3B) of gut microbiota plateaued when the read increased to a certain level. The F and M groups had significantly lower alpha diversity (7.7 and 8.7% lower, respectively, *P* < 0.05) than the MF group (Figure 6A). Adonis analysis showed no noticeable separation between the M and C groups (Figure 6B). However, the fasting groups (F and MF) were clearly separated from the C and M groups (Figure 6B).

LEfSe analysis showed the following metabolic pathway (LDA >2) between each group: secretion system, biosynthesis of unsaturated fatty acid for the F group; phosphotransferase system and other ion coupled transporters for the MF group; membrane and intracellular structural molecules and the citrate cycle TCA cycle for the M group (Figure 6C).

**Figure 7:**
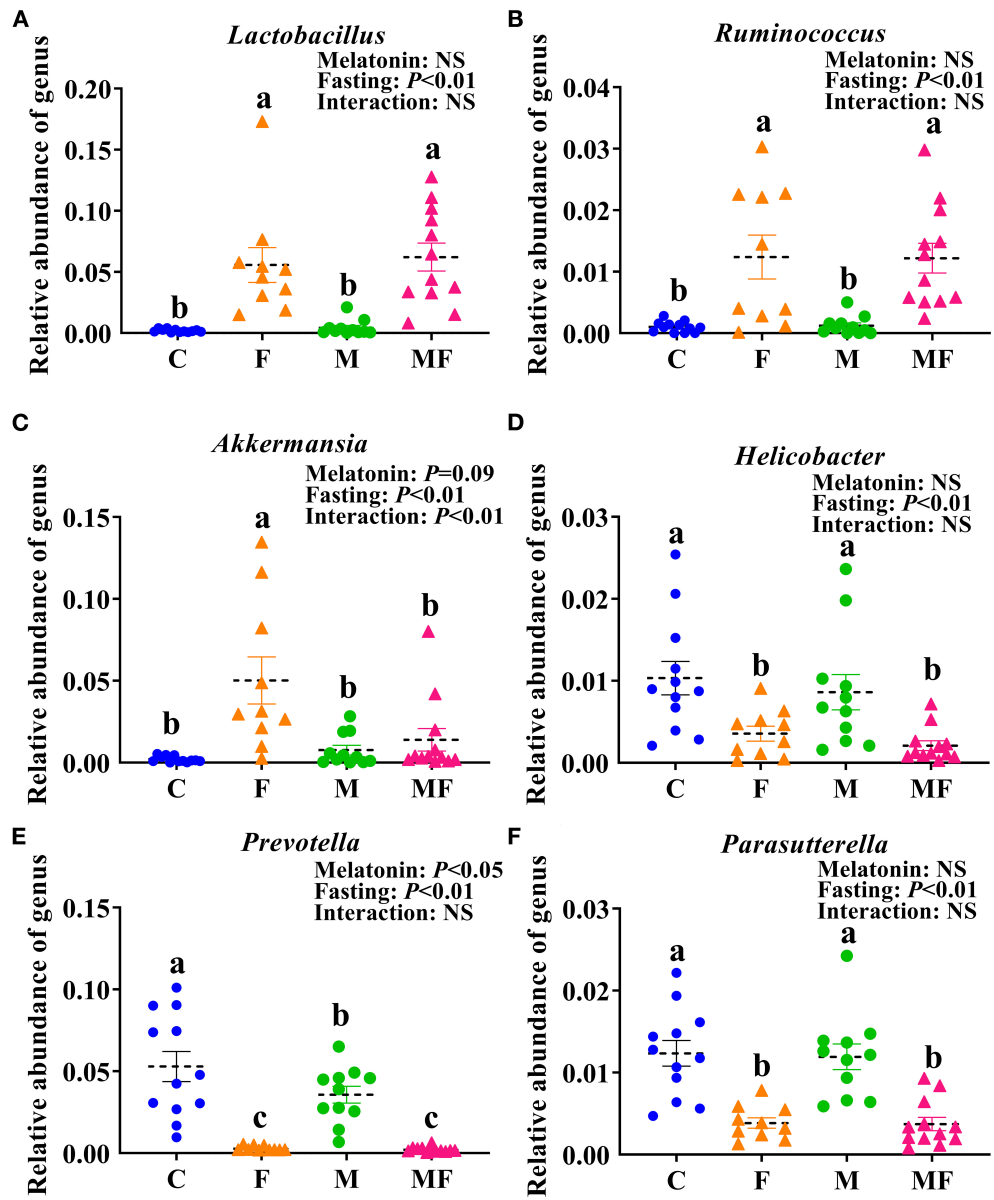
The effects of intermittent fasting and melatonin supplementation on intestinal microbiota at the genus level in mice. Data are presented as mean ± SEM, *n* = 12; Labeled means without a common letter differ (*P* < 0.05), NS: *P* ≥ 0.05. C, control; F, intermittent fasting; M, melatonin; MF, intermittent fasting plus melatonin. The C and M mice were fed *ad libitum*, while the F and MF groups underwent alternative-day feed deprivation. The M and MF groups mice were supplemented with melatonin at a dose of 10 mg/kg body weight by drinking. The relative abundance of *Lactobacillus* **(A)**, *Ruminococcus* **(B)**, *Akkermansia* **(C)**, *Helicobacter* **(D)**, *Prevotella* **(E)**, and *Parasutterella* **(F)** in mice.

There were 32 genera of intestinal flora that were significantly different between the groups. The 6 genera with the most significant difference were shown in Figures 7A–F. Compared with the C group, the F group exhibited a significant increase in the abundance of *Lactobacillus* (fold change, FC = 28.87, *P* < 0.05, Figure 7A), *Ruminococcus* (FC = 12.21, *P* < 0.05, Figure 7B) and *Akkermansia* (FC = 24.19, *P* < 0.05, Figure 7C) and significantly lower abundance of *Helicobacter* (FC = 0.34, *P* < 0.05, Figure 7D), *Prevotella* (FC = 0.05, *P* < 0.05, Figure 7E) and *Parasutterella* (FC = 0.31, *P* < 0.05, Figure 7F). Compared with the C group, the M group had a significantly lower abundance of *Prevotella* (FC = 0.67, *P* < 0.05, Figure 7E). In addition, the interaction between fasting and melatonin had a significant effect on the abundance of *Akkermansia* (*P* < 0.01). Relative abundance by phylum is shown in Supplementary Figure 3C. IF but not melatonin had significant effects on intestinal *Firmicute, Bacteroides* and the radio of *Firmicutes/Bacteroides* (*P* < 0.05, Supplementary Figures 3D–F). In addition, the interaction between fasting and melatonin had a significant effect on the relative abundance of intestinal *Firmicute* and *Bacteroides* (*P* < 0.05).

### Metabolomic Analysis of Intestinal Contents

**Figure 8:**
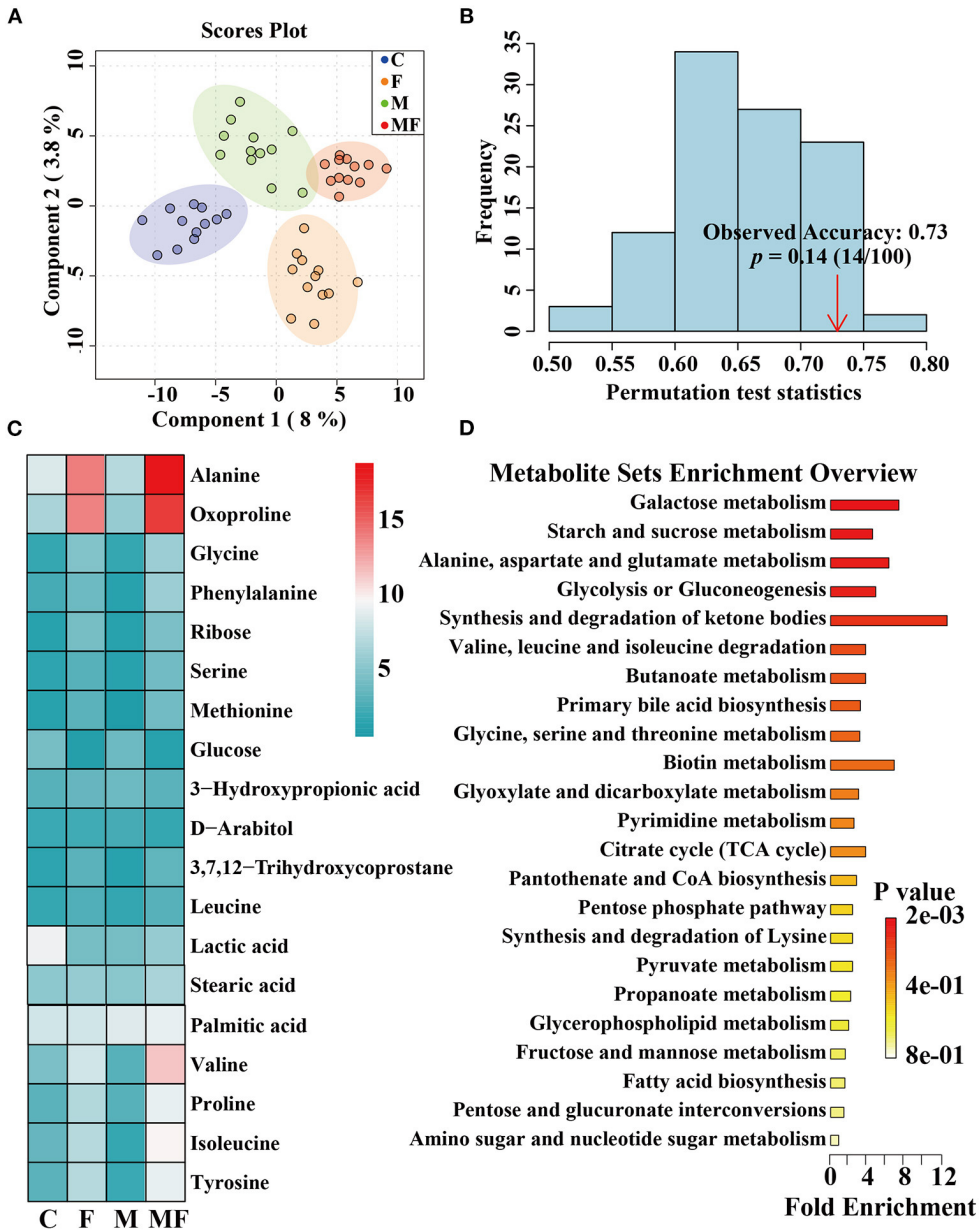
The effects of intermittent fasting and melatonin supplementation on intestinal metabolites in mice. **(A)** Score plot from and PLS-DA of metabolite profiles of the intestinal contents of mice between all groups. Each data point represents a function of the entire spectral profile of each subject; **(B)** The permutation test was evaluated based on the corresponding PLS-DA model. **(C)** A heatmap of significantly altered metabolites in the intestines of mice between all groups. **(D)** Metabolic pathways related to each metabolite. Data are presented as mean ± SEM, *n* = 12. C, control; F, intermittent fasting; M, melatonin; MF, intermittent fasting plus melatonin. The C and M mice were fed *ad libitum*, while the F and MF groups underwent alternative-day feed deprivation. The M and MF groups mice were supplemented with melatonin at a dose of 10 mg/kg body weight by drinking.

The partial least-squares discriminate analysis (PLS-DA) scores plot showed a clear separation between all treatments (Figure 8A). And the permutation test was evaluated based on the corresponding PLS-DA model (Figure 8B). A total of 482 metabolites were detected by metabolomics (Supplementary Table 1) and the top 19 metabolites with the highest abundance were shown in Figure 8C. Differences in metabolites between groups were shown in Supplementary Figure 4. Fasting significantly decreased glucose abundance (*P* < 0.05, Supplementary Figure 4A) but increased ribose, alanine, glycine, valine, isoleucine, tyrosine and 2-ketoadipate abundance (*P* < 0.05, Supplementary Figures 4B–H). Melatonin alone had a significant effect on 2-ketoadipate abundance (*P* < 0.05, Supplementary Figure 4H). The interaction between fasting and melatonin had significant effects on alanine, valine and isoleucine abundances (*P* < 0.05, Supplementary Figures 4C,E,F).

**Figure 9:**
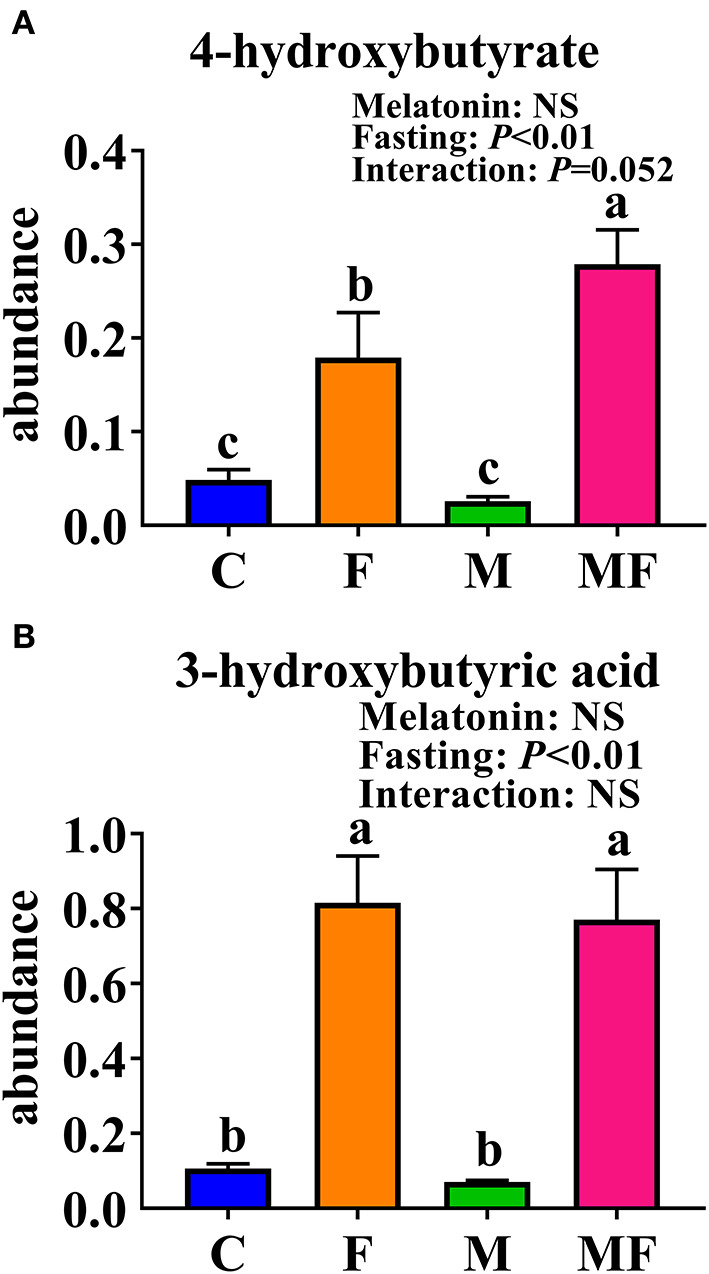
Significantly altered intestinal metabolites between in control mice and those treated with intermittent fasting and melatonin. **(A)** 4-hydroxybutyrate abundance. **(B)** 3-hydroxybutyric acid abundance. Data are presented as mean ± SEM, n = 12; Labeled means without a common letter differ (*P* < 0.05), NS: *P* ≥ 0.05. C, control; F, intermittent fasting; M, melatonin; MF, intermittent fasting plus melatonin. The C and M mice were fed *ad libitum*, while the F and MF groups underwent alternative-day feed deprivation. The M and MF groups mice were supplemented with melatonin at a dose of 10 mg/kg body weight by drinking.

The KEGG enrichment pathways of different metabolites were further analyzed (Figure 8D). The main enrichment pathways were: galactose metabolism; starch and sucrose metabolism, alanine, aspartate and glutamate metabolism, glycolysis or gluconeogenesis, aminoacyl-tRNA biosynthesis and the synthesis and degradation of ketone bodies. Metabolites related to butyric acid metabolism including 4-hydroxybutyrate and 3-hydroxybutyric acid are shown in Figures 9A,B. Fasting had a significant effect on intestinal 4-hydroxybutyrate (Figure 9A) and 3-hydroxybutyric acid (Figure 9B) (*P* < 0.05).

### Correlation Analysis of Phenotype, Bacteria, and Metabolites

We analyzed correlations between the phenotype and the differences of gut microbita or metabolome (Supplementary Figure 5A). Serum glucose and TC levels were positively correlated with the *Prevotella* abundance. The epididymal fat mass and serum glucose level were negatively correlated with the metabolites alanine, isoleucine and methionine.

We also analyzed correlations between differential bacteria (genus) and metabolites (Supplementary Figure 5B). Bacteria from genera *Lactobacillus, Olsenella, Desulfovibrio*, and *Parvibacter* were positively correlated with the metabolites ribose, 3-hydroxybutyric acid, and the amino acids methionine, alanine, isoleucine and valine. However, these metabolites were negatively correlated with the genera *Prevotella, Parasutterella, Parabacteroides, Helicobacter, Paraprevotella, Barnesiella*, and *Vampirovibrio*.

## Discussion

In this study, IF significantly reduced BW of mice, although there was no significant difference in cumulative food intake. IF reduced BW, fat mass, and caloric intake in a manner comparable with a low fat diet (8), suggesting that IF reduces the BW of both obese and normal hosts. A study on IF in obese patients found that weight loss occurs over several weeks because, despite overeating on refeeding days, individuals do not fully compensate for the calorie-deficit realized on fasting days (34). This was similar to what was recorded in our mice model after 24 h of fasting. These effects were in part due to the shift from the utilization of glucose to fatty acids and ketones as the body's preferred fuel source during fasting (35). As shown in this study, fasting groups (F and MF) had a significantly lower serum glucose level, ribose level, and intestinal glucose abundance but significantly higher gut 2-ketoadipate abundance than the C and M groups. Food and water intake in mice seems to follow a pattern of small, frequent meals associated with drinking (36). When the mice were deprived of food on fasting days, they drank significantly less water as well (37). As a result, water intake fluctuated with food intake during fasting.

The morphologic observations of eWAT in the present work demonstrated that fasting resulted in reduced adipocyte area. The number of multilobular adipocytes was significant increased by IF, which is a typical characteristic of beige adipocytes. These results indicate that eWAT browning occurs during fasting. This is consistent with previous reports that found that fasting caused WAT browning in mice, and that IF increased energy expenditure through non-shivering thermogenesis (38). Melatonin was found to directly regulates energy consumption by activating brown adipose tissue and participating in the browning process of WAT (39). Melatonin had a significant effect on the number of adipocytes per square millimeter in the present work, but no effect on adipocyte size.

Several studies have shown that melatonin prevents obesity in different animal models (18, 40, 41) and has a protective effect against dietary-induced obesity (18). In this study, oral administration of melatonin through the drinking water improved serum melatonin level but had no effect on body weight gain and serum indexes of mice fed on normal diet. The level of serum melatonin was positively correlated with the abundance of *Allobaculum*, indicating that the addition of exogenous melatonin may affect the content of *Allobaclum* in the intestine.

Intestinal morphology can be used to assess intestine function and health (42). The long villi and shallow crypts of the ileum are associated with digestive and absorptive function (43). In this study, the F group had significantly greater ileal villi length and villi length/crypt depth than the C group, indicating that the ileal villi became longer and the crypts became shallower to enhance the digestive and absorptive capacity of the small intestine in order to adapt to the relative lack of nutrition under fasting conditions.

Fasting was able to induce the accumulation of lipid droplets all over cells (44). Lipid droplets rich in triglycerides are efficiently decomposed in hepatocytes to provide fatty acids for the formation of lipoprotein particles (45). In this study, fasting may lead to the influx of fatty acids from adipose tissue into the liver to a certain extent, thereby increasing the number of lipid droplets in the liver.

Studies on obese mice have shown that the obese microbiome has an increased capacity to harvest energy from the diet (46). The obese mice fed with HFD had significantly higher *Firmicutes* but lower *Bacteroides* relative abundance in caecum (47). In this study, IF changed the gut microbiota, with an increased abundance of *Firmicutes* but a decreased abundance of *Bacteroides*, which eventually led to dramatic changes in microbiota composition and an increased *Firmicutes/Bacteroidetes* ratio. It indicated that IF changed the intestinal microbiota of mice and led them similar to obesity, that is to say, IF increased the ability of intestinal microbiota to obtain energy from diet. This finding are consistent with the results of previous study (38). Similar shifts in *Firmicutes/Bacteroidetes* ratio in mice were found to be associated with enhanced energy extraction during cold stress and increased glucose uptake in inguinal WAT (48).

The vast majority of butyrate-producing bacteria in the gut is *Firmicutes* (49), and butyric acid absorbed by the colonic mucosa is the preferred energy source for colonocytes (50). Metabonomic analysis of intestinal contents showed that fasting increased the 3-hydroxybutyric acid and 4-hydroxybutyric acid content, which may correspond with changes in microorganisms.

IF increased the abundance of lactic acid bacteria. *Lactobacillus* has the ability to digest nutrients and may have a significant influence on carbohydrate metabolism (51). This study showed that the abundance of intestinal *Lactobacillus* was negatively correlated with serum glucose level, TC and body weight gain. And the abundance of *Lactobacillus* was positively correlated with the contents of various metabolites (3-hydroxybutyric acid, ribose, 3, 7, 12-trihydroxycoprostane). IF induced enrichment of *Lactobacilli*, which are commonly used as probiotics because of their beneficial effects, including reduction of inflammatory immune responses (52). *Akkermansia* also increased under fasting conditions in this study. *A. muciniphila* not only participates in the host immune regulation, but also enhances the integrity of the intestinal epithelial cells and the thickness of the mucus layer, thereby promoting intestinal health (53, 54). In this study, fasting led to a significantly higher abundance of *Akkermansia* in the gut of mice compared with un-fasting treatment (the C and M group), which indicating that the nutritional supply of the host could affect the growth of *A. muciniphila* in the intestine. When the host is under the fasting or in malnutrition, *A. muciniphila* revealing the characteristics of degrading mucin can be defined as a competitive advantage, which was consistent with the experiment on hamsters that the abundance of *A. muciniphila* significantly increased after fasting (55).

In the metagenomic analysis, the ketone pathway was enhanced in the gut microbiome of IF mice, which suggests that the gut microbiome regulates its own ketone body metabolism during fasting of the host (56). Ketones are the preferred fuel for the brain and body during fasting, so the body prioritizes ketones that are converted from the glycogen decomposition of glucose to fatty acids (57). Fasting increased the abundance of 2-ketoadipate in the intestine in the present work, and metabolites related to the synthesis and degradation of ketone bodies were significantly increased. This shift indicates that the body has shifted from lipid synthesis and fat storage to mobilizing fat in the form of free fatty acids and fatty acid-derived ketones (35). We found that fasting changed the energy intake pattern in mice, which led to changes in metabolism. Metabonomic analysis of mice intestinal contents showed that the metabolic changes caused by IF were mainly reflected by the relative levels of sugars, amino acids and fatty acids.

In conclusion, IF reduced BW gain, serum glucose, TC, TG, insulin level, and adipocyte size, then increased the number of adipocyte per square millimeter. IF also resulted in increased ileal villus height to optimize digestion and absorption, thereby modulating the intestinal microbiota and metabolites. Melatonin alone had no effect, and there was little interaction between IF and melatonin except for their effects on adipocyte area and number, the abundance of *Bacteroidetes* and *Akkermansia*, and the intestinal metabolites alanine, valine and isoleucine. These results indicate that IF may alter metabolism, reduce obesity and improve intestinal health.

## Data Availability Statement

The datasets presented in this study can be found in online repositories. The names of the repository/repositories and accession number(s) can be found at: https://www.ncbi.nlm.nih.gov/, PRJNA766780.

## Ethics Statement

The animal study was reviewed and approved by Animal Care and Use Committee of Zhejiang University (ETHICS CODE Permit No. ZJU20170529).

## Author Contributions

HW designed the experiments. JinL, YZ, and YM performed the experiments. JinL, HW, and YZ analyzed the data. HW, JinL, XL, and JiaL wrote and revised the main manuscript. All authors read and approved the final manuscript.

## Funding

The study was supported by grants from the Natural Science Foundation of Zhejiang Province (Z19C170001), the National Natural Science Foundation of China (31672430), the Funds of Ten Thousand People Plan of China and the National Key Research and Development Program of China (2017YFD0500502).

## Conflict of Interest

The authors declare that the research was conducted in the absence of any commercial or financial relationships that could be construed as a potential conflict of interest.

## Publisher's Note

All claims expressed in this article are solely those of the authors and do not necessarily represent those of their affiliated organizations, or those of the publisher, the editors and the reviewers. Any product that may be evaluated in this article, or claim that may be made by its manufacturer, is not guaranteed or endorsed by the publisher.

## Supplementary Material

Supplementary Figure 1 Changes in the food, water, and melatonin intake of mice treated with intermittent fasting and melatonin. *n* = 12. (1C) The abscissa indicates that a non-fasting and fasting day is a cycle. C, control; F, intermittent fasting; M, melatonin; MF, intermittent fasting plus melatonin. The C and M mice were fed *ad libitum*, while the F and MF groups underwent alternative-day feed deprivation. The M and MF groups mice were supplemented with melatonin at a dose of 10 mg/kg body weight by drinking. Black arrows indicate fasting days.

Supplementary Figure 2 Villus height and crypt depth of the ilea and the colonic folds of colon in control mice and those treated with intermittent fasting and melatonin. **(A)** Representative H&E staining of ileum sections. Scale bar: 50 μm; **(B)** representative H&E staining of colon sections. Scale bar: 100 μm; **(C)** Villus height, crypt depth and villus/crypt ratio of ileum between groups; **(D)** Colonic folds between groups. Villus height, crypt depth and the length of colonic fold were measured as indicated in the image. Data are presented as mean ± SEM, *n* = 6; Labeled means without a common letter differ (*P* < 0.05), NS: *P* ≥ 0.05. C, control; F, intermittent fasting; M, melatonin; MF, intermittent fasting plus melatonin. The C and M mice were fed *ad libitum*, while the F and MF groups underwent alternative-day feed deprivation. The M and MF groups mice were supplemented with melatonin at a dose of 10 mg/kg body weight by drinking.

Supplementary Figure 3 The effects of intermittent fasting and melatonin supplementation on intestinal microbiota of mice. **(A)** The observed species index of gut microbiota in mice. **(B)** The Chao 1 index of gut microbiota in mice. **(C)** Barplot of intestinal bacterial content at the phylum level in each group; **(D)** relative abundance of *Firmicutes* in the intestinal contents of each group; **(E)** relative abundance of *Bacteroides* in the intestinal contents of each group; **(F)** the proportion of *Firmicutes* to *Bacteroides*. Data are presented as mean ± SEM, *n* = 12; Labeled means without a common letter differ (*P* < 0.05), NS: *P* ≥ 0.05. C, control; F, intermittent fasting; M, melatonin; MF, intermittent fasting plus melatonin. The C and M mice were fed *ad libitum*, while the F and MF groups underwent alternative-day feed deprivation. The M and MF groups mice were supplemented with melatonin at a dose of 10 mg/kg body weight by drinking.

Supplementary Figure 4 Significantly altered intestinal metabolites between in control mice and those treated with intermittent fasting and melatonin. The contents of glucose **(A)**, ribose **(B)**, alanine **(C)**, glycine **(D)**, valine **(E)**, isoleucine **(F)**, tyrosine **(G)**, and 2-ketoadipate **(H)** in each group are shown. Data are presented as mean ± SEM, *n* = 12; Labeled means without a common letter differ (*P* < 0.05), NS: *P* ≥ 0.05. C, control; F, intermittent fasting; M, melatonin; MF, intermittent fasting plus melatonin. The C and M mice were fed *ad libitum*, while the F and MF groups underwent alternative-day feed deprivation. The M and MF groups mice were supplemented with melatonin at a dose of 10 mg/kg body weight by drinking.

Supplementary Figure 5 Correlations between the phenotype and the differences of bacteria (genus) or metabolites in the intestine. **(A)** Correlations between the phenotype and the differences of bacteria (genus) and metabolites. **(B)** Correlations between bacteria (genus) and metabolites in the intestine.

Supplementary Table 1 All the metabolites detected in the intestinal contents in mice. C, control; F, intermittent fasting; M, melatonin; MF, intermittent fasting plus melatonin. The C and M mice were fed *ad libitum*, while the F and MF groups underwent alternative-day feed deprivation. The M and MF groups mice were supplemented with melatonin at a dose of 10 mg/kg body weight by drinking.

## Abbreviations

ALT: alanine aminotransferase
AST: aspartate aminotransferase
eWAT: epididymal white adipose tissue
FC: fold change
FDR: false discovery rate
Glu: glucose
HDL-C: high density lipoproteins cholesterol
IF: Intermittent fasting
KEGG: Kyoto Encyclopedia of Genes and Genomes
LDA: linear discriminant analysis
LDL-C: low density lipoproteins cholesterol
OTU: operational taxonomic units
PCoA: principal component analysis
PLS-DA: partial least squares-discriminant analysis
RT: retention time
TC: total cholesterol
TEM: Transmission electron microscopy
TG: triglyceride
VIP: variable importance in projection.

## References

[1] Inoue KI Toyoda S Jojima T Abe S Sakuma M Inoue T. Time-restricted feeding prevents high-fat and high-cholesterol diet-induced obesity but fails to ameliorate atherosclerosis in apolipoprotein E-knockout mice. Exp Anim. (2020) 70:194–202. 10.1538/expanim.20-0112 33268668 PMC8150245

[2] Singh GM Danaei G Farzadfar F Stevens GA Woodward M Wormser D. The age-specific quantitative effects of metabolic risk factors on cardiovascular diseases and diabetes: a pooled analysis. PLoS ONE. (2013) 8:e65174. 10.1371/journal.pone.0065174 23935815 PMC3728292

[3] Lauby-Secretan B Scoccianti C Loomis D Grosse Y Bianchini F Straif K. Body fatness and cancer—viewpoint of the IARC working group. N Engl J Med. (2016) 375:794–8. 10.1056/NEJMsr1606602 27557308 PMC6754861

[4] Hill JO Wyatt HR Peters JC. Energy balance and obesity. Circulation. (2012) 126:126–32. 10.1161/CIRCULATIONAHA.111.087213 22753534 PMC3401553

[5] Patterson RE Laughlin GA LaCroix AZ Hartman SJ Natarajan L Senger CM. Intermittent fasting and human metabolic health. J Acad Nutr Diet. (2015) 115:1203–12. 10.1016/j.jand.2015.02.018 25857868 PMC4516560

[6] Eshghinia S Mohammadzadeh F. The effects of modified alternate-day fasting diet on weight loss and CAD risk factors in overweight and obese women. J Diabetes Metab Disord. (2013) 12:4. 10.1186/2251-6581-12-4 23497604 PMC3598220

[7] Lopez-Bueno M Gonzalez-Jimenez E Navarro-Prado S Montero-Alonso MA Schmidt-RioValle J. Influence of age and religious fasting on the body composition of Muslim women living in a westernized context. Nutr Hosp. (2014) 31:1067–73. 10.3305/nh.2015.31.3.8278 25726194

[8] Gotthardt JD Verpeut JL Yeomans BL Yang JA Yasrebi A Roepke TA. Intermittent fasting promotes fat loss with lean mass retention, increased hypothalamic norepinephrine content, and increased neuropeptide Y gene expression in diet-induced obese male mice. Endocrinology. (2016) 157:679–91. 10.1210/en.2015-1622 26653760 PMC4733124

[9] Mattson MP. Emerging neuroprotective strategies for Alzheimer's disease: dietary restriction, telomerase activation, and stem cell therapy. Exp Gerontol. (2000) 35:489–502. 10.1016/S0531-5565(00)00115-7 10959037

[10] Mattson MP Allison DB Fontana L Harvie M Longo VD Malaisse WJ. Meal frequency and timing in health and disease. Proc Natl Acad Sci USA. (2014) 111:16647–53. 10.1073/pnas.1413965111 25404320 PMC4250148

[11] Panda S. Circadian physiology of metabolism. Science. (2016) 354:1008–15. 10.1126/science.aah4967 27885007 PMC7261592

[12] Andrea DF Clara DG Michel B Rafael dC. A time to fast. Science. (2018) 362:770–5. 10.1126/science.aau2095 30442801 PMC8504313

[13] Auld F Maschauer EL Morrison I Skene DJ Riha RL. Evidence for the efficacy of melatonin in the treatment of primary adult sleep disorders. Sleep Med Rev. (2017) 34:10–22. 10.1016/j.smrv.2016.06.005 28648359

[14] Acuna-Castroviejo D Escames G Venegas C Diaz-Casado ME Lima-Cabello E Lopez LC. Extrapineal melatonin: sources, regulation, and potential functions. Cell Mol Life Sci. (2014) 71:2997–3025. 10.1007/s00018-014-1579-2 24554058 PMC11113552

[15] Ma N Zhang J Reiter RJ Ma X. Melatonin mediates mucosal immune cells, microbial metabolism, and rhythm crosstalk: a therapeutic target to reduce intestinal inflammation. Med Res Rev. (2020) 40:606–32. 10.1002/med.21628 31420885

[16] Tan DX Liu X Manchester LC Rosales-Corral SA Reiter RJ. Melatonin in the biliary tract and liver: health implications. Curr Pharm Des. (2014) 20:4788–801. 10.2174/1381612819666131119105826 24251672

[17] Amaral FGD Andrade-Silva J Kuwabara WMT Cipolla-Neto J. New insights into the function of melatonin and its role in metabolic disturbances. Expert Rev Endocrinol Metab. (2019) 14:293–300. 10.1080/17446651.2019.1631158 31192707

[18] Xu PF Wang JL Hong F Wang S Jin X Xue TT. Melatonin prevents obesity through modulation of gut microbiota in mice. J Pineal Res. (2017) 62:e12399. 10.1111/jpi.12399 28199741

[19] Huether G. Melatonin synthesis in the gastrointestinal tract and the impact of nutritional factors on circulating melatonin. Ann NY Acad Sci. (1994) 719:146–58. 10.1111/j.1749-6632.1994.tb56826.x 8010590

[20] Gil-Martin E Egea J Reiter RJ Romero A. The emergence of melatonin in oncology: focus on colorectal cancer. Med Res Rev. (2019) 39:2239–85. 10.1002/med.21582 30950095

[21] Heinsen FA Knecht H Neulinger SC Schmitz RA Knecht C Kuhbacher T. Dynamic changes of the luminal and mucosa-associated gut microbiota during and after antibiotic therapy with paromomycin. Gut Microbes. (2015) 6:243–54. 10.1080/19490976.2015.1062959 26178862 PMC4615565

[22] Forsythe P Kunze WA Bienenstock J. On communication between gut microbes and the brain. Curr Opin Gastroenterol. (2012) 28:557–62. 10.1097/MOG.0b013e3283572ffa 23010679

[23] Paulose JK Cassone VM. The melatonin-sensitive circadian clock of the enteric bacterium Enterobacter aerogenes. Gut Microbes. (2016) 7:424–7. 10.1080/19490976.2016.1208892 27387841 PMC5154366

[24] Lin CJ Chiu CC Chen YC Chen ML Hsu TC Tzang BS. Taurine attenuates hepatic inflammation in chronic alcohol-fed rats through inhibition of TLR4/MyD88 signaling. J Med Food. (2015) 18:1291–8. 10.1089/jmf.2014.3408 26090712 PMC4685501

[25] Liu Z Dai X Zhang H Shi R Hui Y Jin X. Gut microbiota mediates intermittent-fasting alleviation of diabetes-induced cognitive impairment. Nat Commun. (2020) 11:855–68. 10.1038/s41467-020-14676-4 32071312 PMC7029019

[26] Yildirim A Arabaci Tamer S Sahin D Bagriacik F Kahraman MM Onur ND. The effects of antibiotics and melatonin on hepato-intestinal inflammation and gut microbial dysbiosis induced by a short-term high-fat diet consumption in rats. Br J Nutr. (2019) 122:841–55. 10.1017/S0007114519001466 31217044

[27] Fadrosh DW Ma B Gajer P Sengamalay N Ott S Brotman RM. An improved dual-indexing approach for multiplexed 16S rRNA gene sequencing on the Illumina MiSeq platform. Microbiome. (2014) 2:6–12. 10.1186/2049-2618-2-6 24558975 PMC3940169

[28] Edgar RC. UPARSE. highly accurate OTU sequences from microbial amplicon reads. Nat Methods. (2013) 10:996–8. 10.1038/nmeth.2604 23955772

[29] Langille MG Zaneveld J Caporaso JG McDonald D Knights D Reyes JA. Predictive functional profiling of microbial communities using 16S rRNA marker gene sequences. Nat Biotechnol. (2013) 31:814–21. 10.1038/nbt.2676 23975157 PMC3819121

[30] Gao K Liu L Dou XX Wang C Liu JX Zhang WM. Doses Lactobacillus reuteri depend on adhesive ability to modulate the intestinal immune response and metabolism in mice challenged with lipopolysaccharide. Sci Rep. (2016) 6:28332–43. 10.1038/srep28332 27323686 PMC4915000

[31] Kind T Wohlgemuth G Lee DY Lu Y Palazoglu M Shahbaz S. FiehnLib: mass spectral and retention index libraries for metabolomics based on quadrupole and time-of-flight gas chromatography/mass spectrometry. Anal Chem. (2009) 81:10038–48. 10.1021/ac9019522 19928838 PMC2805091

[32] Dunn WB Broadhurst D Begley P Zelena E Francis-McIntyre S Anderson N. Procedures for large-scale metabolic profiling of serum and plasma using gas chromatography and liquid chromatography coupled to mass spectrometry. Nat Protoc. (2011) 6:1060–83. 10.1038/nprot.2011.335 21720319

[33] Chong J Soufan O Li C Caraus I Li S Bourque G. MetaboAnalyst 4.0: towards more transparent and integrative metabolomics analysis. Nucleic Acids Res. (2018) 46:W486–94. 10.1093/nar/gky310 29762782 PMC6030889

[34] Klempel MC Kroeger CM Varady KA. Alternate day fasting (ADF) with a high-fat diet produces similar weight loss and cardio-protection as ADF with a low-fat diet. Metabolism. (2013) 62:137–43. 10.1016/j.metabol.2012.07.002 22889512

[35] Anton SD Moehl K Donahoo WT Marosi K Lee SA Mainous AG 3rd. Flipping the metabolic switch: understanding and applying the health benefits of fasting. Obesity. (2018) 26:254–68. 10.1002/oby.22065 29086496 PMC5783752

[36] Jensen TL Kiersgaard MK Sorensen DB Mikkelsen LF. Fasting of mice: a review. Lab Anim. (2013) 47:225–40. 10.1177/0023677213501659 24025567

[37] Kurokawa M Akino K Kanda KA. New apparatus for studying feeding and drinking in the mouse. Physiol Behav. (2000) 70:105–12. 10.1016/S0031-9384(00)00226-2 10978484

[38] Li G Xie C Lu S Nichols RG Tian Y Li L. Intermittent fasting promotes white adipose browning and decreases obesity by shaping the gut microbiota. Cell metabolism. (2017) 26:672–85.e4. 10.1016/j.cmet.2017.08.019 28918936 PMC5668683

[39] Cipolla-Neto J Amaral FG Afeche SC Tan DX Reiter RJ. Melatonin, energy metabolism, and obesity: a review. J Pineal Res. (2014) 56:371–81. 10.1111/jpi.12137 24654916

[40] Ríos-Lugo MJ Cano P Jiménez-Ortega V Fernández-Mateos MP Scacchi PA Cardinali DP. Melatonin effect on plasma adiponectin, leptin, insulin, glucose, triglycerides and cholesterol in normal and high fat-fed rats. J Pineal Res. (2010) 49:342–8. 10.1111/j.1600-079X.2010.00798.x 20663045

[41] Prunet-Marcassus B Desbazeille M Bros A Louche K Delagrange P Renard P. Melatonin reduces body weight gain in Sprague Dawley rats with diet-induced obesity. Endocrinology. (2003) 144:5347–52. 10.1210/en.2003-0693 12970162

[42] Liao SF Nyachoti M. Using probiotics to improve swine gut health and nutrient utilization. Anim Nutr. (2017) 3:331–43. 10.1016/j.aninu.2017.06.007 29767089 PMC5941265

[43] Pluske JR Williams IH Aherne FX. Villous height and crypt depth in piglets in response to increases in the intake of cows' milk after weaning. Anim Sci. (1996) 62:145–58. 10.1017/S135772980001442930886898

[44] Rai P Kumar M Sharma G Barak P Das S Kamat SS. Kinesin-dependent mechanism for controlling triglyceride secretion from the liver. Proc Natl Acad Sci USA. (2017) 114:12958–63. 10.1073/pnas.1713292114 29158401 PMC5724275

[45] Kumar M Ojha S Rai P Joshi A Kamat SS Mallik R. Insulin activates intracellular transport of lipid droplets to release triglycerides from the liver. J Cell Biol. (2019) 218:3697–713. 10.1083/jcb.201903102 31604801 PMC6829650

[46] Turnbaugh PJ Ley RE Mahowald MA Magrini V Mardis ER Gordon JI. An obesity-associated gut microbiome with increased capacity for energy harvest. Nature. (2006) 444:1027–31. 10.1038/nature05414 17183312

[47] Turnbaugh PJ Bäckhed F Fulton L Gordon JI. Diet-induced obesity is linked to marked but reversible alterations in the mouse distal gut microbiome. Cell Host Microbe. (2008) 3:213–23. 10.1016/j.chom.2008.02.015 18407065 PMC3687783

[48] Chevalier C Stojanovic O Colin DJ Suarez-Zamorano N Tarallo V Veyrat-Durebex C. Gut microbiota orchestrates energy homeostasis during cold. Cell. (2015) 163:1360–74. 10.1016/j.cell.2015.11.004 26638070

[49] Fu X Liu Z Zhu C Mou H Kong Q. Nondigestible carbohydrates, butyrate, and butyrate-producing bacteria. Crit Rev Food Sci Nutr. (2019) 59:S130–52. 10.1080/10408398.2018.1542587 30580556

[50] Clausen MR Mortensen PB. Kinetic studies on colonocyte metabolism of short chain fatty acids and glucose in ulcerative colitis. Gut. (1995) 37:684–9. 10.1136/gut.37.5.684 8549946 PMC1382875

[51] Drissi F Raoult D Merhej V. Metabolic role of lactobacilli in weight modification in humans and animals. Microb Pathog. (2017) 106:182–94. 10.1016/j.micpath.2016.03.006 27033001

[52] Umbrello G Esposito S. Microbiota and neurologic diseases: potential effects of probiotics. J Transl Med. (2016) 14:298. 10.1186/s12967-016-1058-7 27756430 PMC5069982

[53] Everard A Belzer C Geurts L Ouwerkerk JP Druart C Bindels LB. Cross-talk between *Akkermansia muciniphila* and intestinal epithelium controls diet-induced obesity. Proc Natl Acad Sci USA. (2013) 110:9066–71. 10.1073/pnas.1219451110 23671105 PMC3670398

[54] Reunanen J Kainulainen V Huuskonen L Ottman N Belzer C Huhtinen H. *Akkermansia muciniphila* adheres to enterocytes and strengthens the integrity of the epithelial cell layer. Appl Environ Microbiol. (2015) 81:3655–62. 10.1128/AEM.04050-14 25795669 PMC4421065

[55] Sonoyama K Fujiwara R Takemura N Ogasawara T Watanabe J Ito H. Response of gut microbiota to fasting and hibernation in Syrian hamsters. Appl Environ Microbiol. (2009) 75:6451–6. 10.1128/AEM.00692-09 19700553 PMC2765128

[56] Cignarella F Cantoni C Ghezzi L Salter A Dorsett Y Chen L. Intermittent fasting confers protection in CNS autoimmunity by altering the gut microbiota. Cell Metabol. (2018) 27:1222–35.e6. 10.1016/j.cmet.2018.05.006 29874567 PMC6460288

[57] Puchalska P Crawford PA. Multi-dimensional roles of ketone bodies in fuel metabolism, signaling, and therapeutics. Cell Metab. (2017) 25:262–84. 10.1016/j.cmet.2016.12.022 28178565 PMC5313038

