# Supplementary figures and images for: Intermittent Fasting Reshapes the Gut Microbiota and Metabolome and Reduces Weight Gain More Effectively Than Melatonin in Mice
Source: Front Nutr. 2021 Nov 24;8:784681. doi: 10.3389/fnut.2021.784681 (PMC8652062; doi:10.3389/fnut.2021.784681)

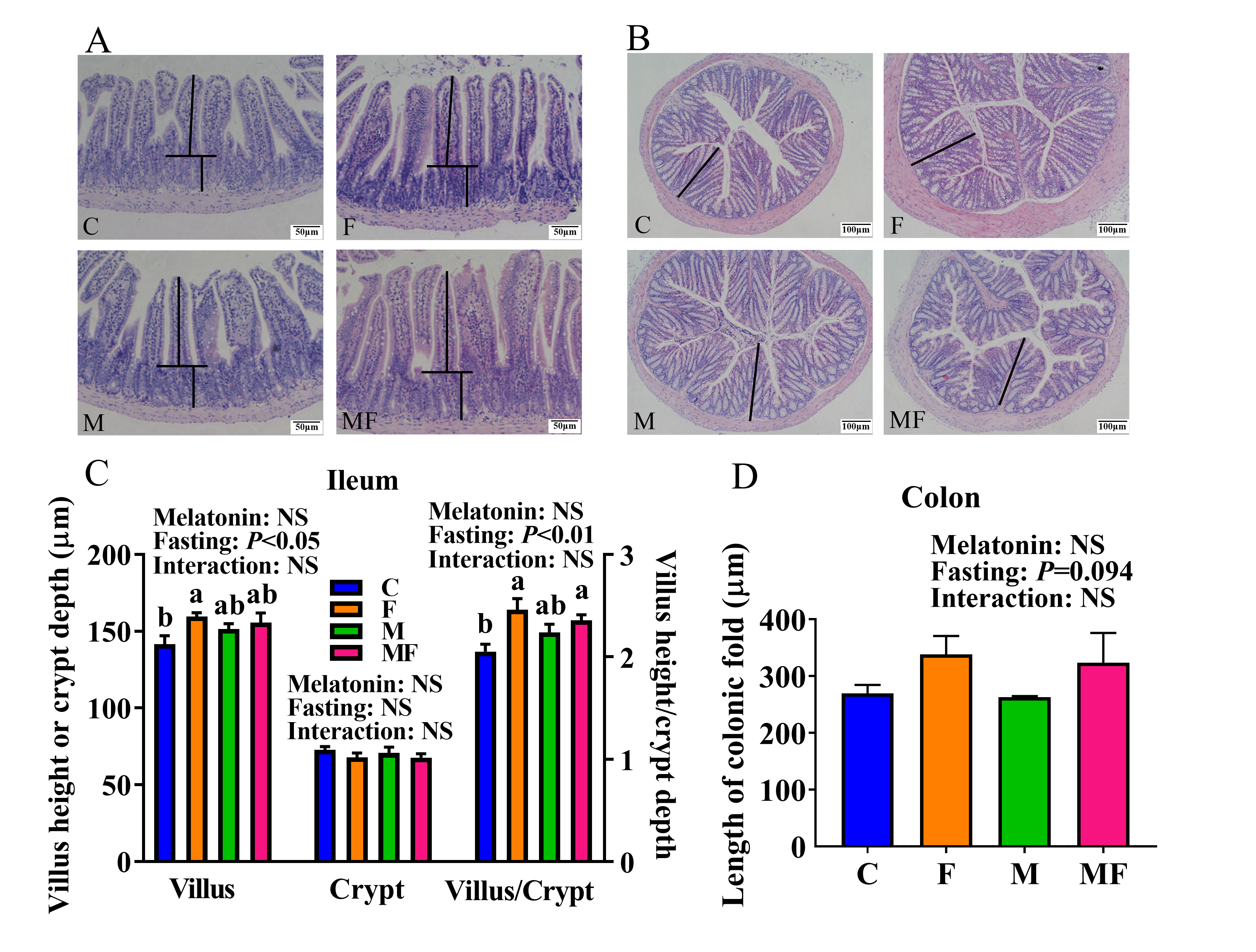

Supplement: Supplementary Figure 2 — Villus height and crypt depth of the ilea and the colonic folds of colon in control mice and those treated with intermittent fasting and melatonin. (A) Representative H&E staining of ileum sections. Scale bar: 50 μm; (B) representative H&E staining of colon sections. Scale bar: 100 μm; (C) Villus height, crypt depth and villus/crypt ratio of ileum between groups; (D) Colonic folds between groups. Villus height, crypt depth and the length of colonic fold were measured as indicated in the image. Data are presented as mean ± SEM, n = 6; Labeled means without a common letter differ (P < 0.05), NS: P ≥ 0.05. C, control; F, intermittent fasting; M, melatonin; MF, intermittent fasting plus melatonin. The C and M mice were fed ad libitum, while the F and MF groups underwent alternative-day feed deprivation. The M and MF groups mice were supplemented with melatonin at a dose of 10 mg/kg body weight by drinking. [file Image_2.tif]

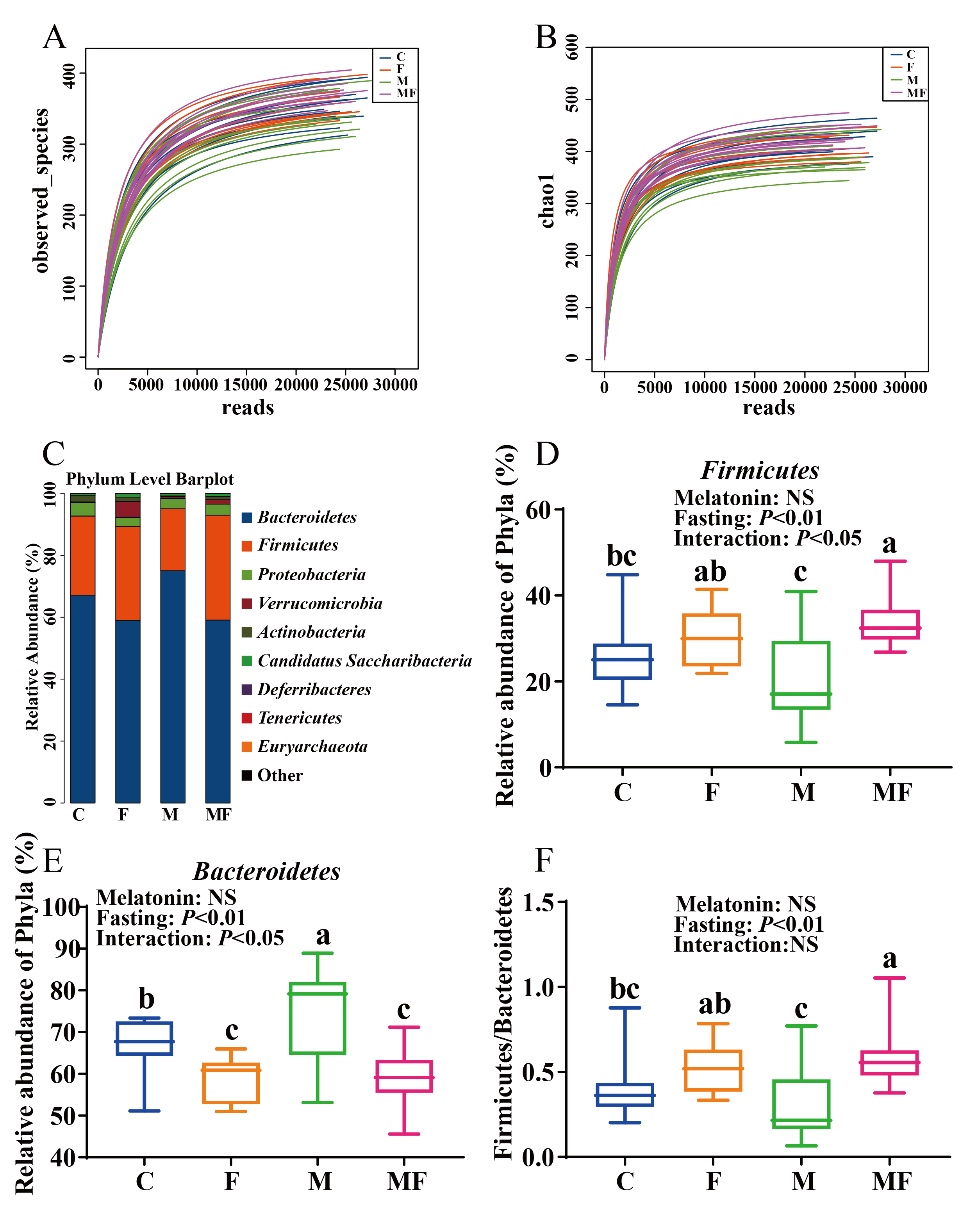

Supplement: Supplementary Figure 3 — The effects of intermittent fasting and melatonin supplementation on intestinal microbiota of mice. (A) The observed species index of gut microbiota in mice. (B) The Chao 1 index of gut microbiota in mice. (C) Barplot of intestinal bacterial content at the phylum level in each group; (D) relative abundance of Firmicutes in the intestinal contents of each group; (E) relative abundance of Bacteroides in the intestinal contents of each group; (F) the proportion of Firmicutes to Bacteroides. Data are presented as mean ± SEM, n = 12; Labeled means without a common letter differ (P < 0.05), NS: P ≥ 0.05. C, control; F, intermittent fasting; M, melatonin; MF, intermittent fasting plus melatonin. The C and M mice were fed ad libitum, while the F and MF groups underwent alternative-day feed deprivation. The M and MF groups mice were supplemented with melatonin at a dose of 10 mg/kg body weight by drinking. [file Image_3.tif]

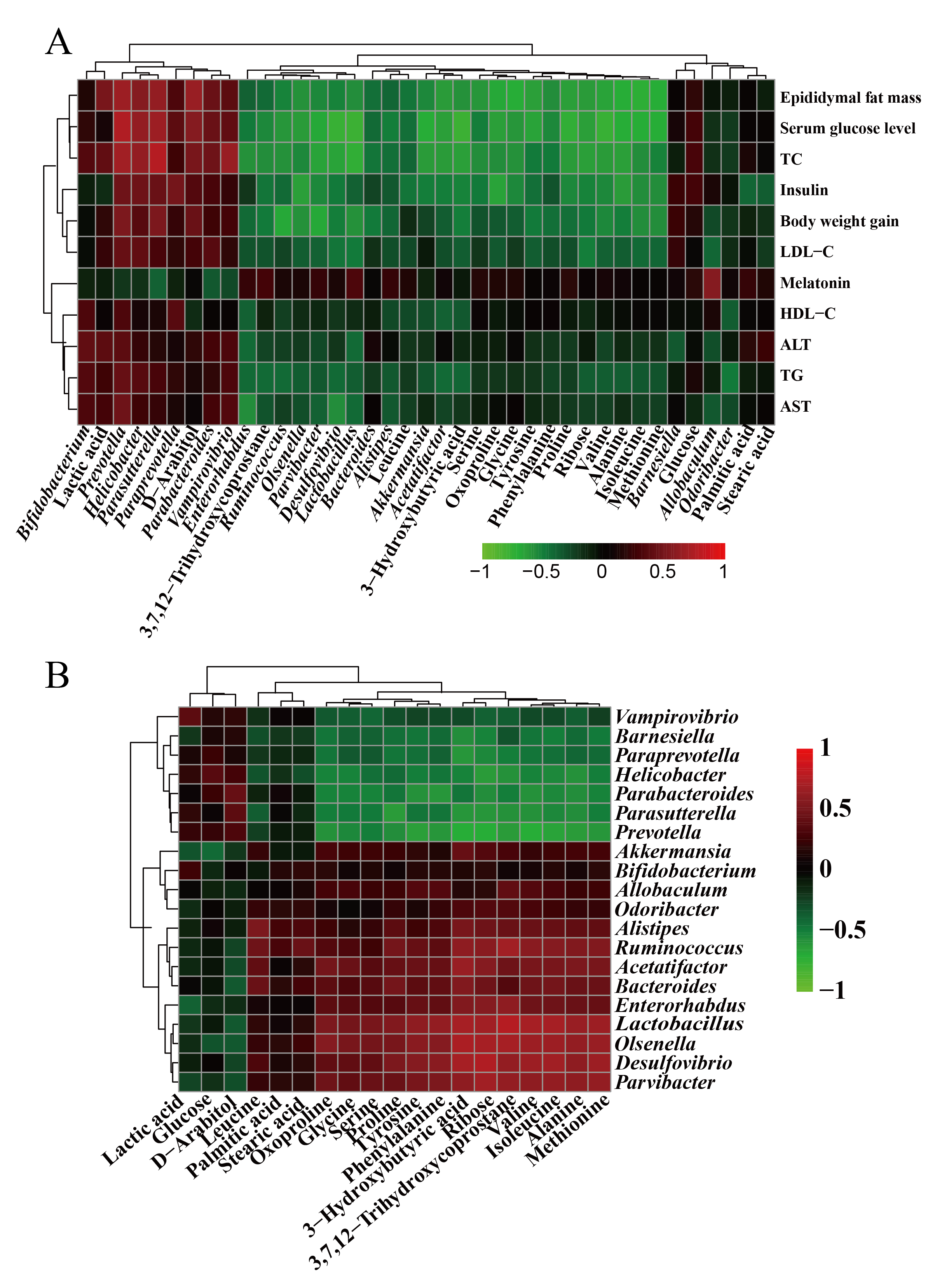

Supplement: Supplementary Figure 5 — Correlations between the phenotype and the differences of bacteria (genus) or metabolites in the intestine. (A) Correlations between the phenotype and the differences of bacteria (genus) and metabolites. (B) Correlations between bacteria (genus) and metabolites in the intestine. [file Image_5.tif]
